# Interrelationships between malnutrition, dehydration, frailty, and sarcopenia in older adults with proximal femur fractures: a prospective observational study

**DOI:** 10.1007/s40520-026-03364-w

**Published:** 2026-04-13

**Authors:** Belinda Trobec, Gianluca Canton, Emma Spedicato, Eleonora Wabitsch, Paolo De Colle, Michela Zanetti, Andrea Marchetti, Luigi Murena, Chiara Ratti

**Affiliations:** 1 Department of Medicine, Surgery and Health Sciences, University of Trieste, Strada di Fiume 447, Trieste, 34149 Italy; 2 Azienda Sanitaria Universitaria Giuliano Isontina, Strada di Fiume 447, Trieste, 34149 Italy; 3 Orthopedics and Traumatology, Cattinara Hospital, Strada di Fiume 447, Trieste, 34149 Italy; 4 Geriatric Department, Hospital Maggiore, Trieste, Italy

**Keywords:** geriatrics, hip fracture, malnutrition, dehydration, sarcopenia, mortality, frailty

## Abstract

**Background:**

Proximal femur fractures (PFFs) are a hallmark of frailty in patients aged ≥ 65 years. Geriatric frailty is multifactorial, with malnutrition, dehydration, and sarcopenia negatively impacting outcomes after PFF.

**Aims:**

The primary aim of this study was to examine the interrelationships between frailty, malnutrition, hydration status, and ultrasound-defined muscle impairment in older patients with proximal femur fracture. As a secondary objective, we explored the association of these vulnerability domains with 3-month mortality as an exploratory outcome.

**Methods:**

In this prospective observational study, 108 consecutive patients aged ≥ 65 years undergoing surgical treatment for PFF at Cattinara Hospital (Trieste) were evaluated. Clinical, nutritional (MNA, MUST), functional (ADL, CFS, NHFS, MPI), cognitive (SPMSQ), and dehydration (CDS) assessments were performed. Ultrasound sarcopenia index (USI) measurements were obtained in both upper and lower limbs of 78 patients.

**Results:**

Mean age was 85.9 ± 6.6 years. Malnutrition, clinical dehydration, and frailty were present in 2.7%, 38%, and 56% of patients, respectively. Among patients assessed by ultrasound, 39% showed sarcopenic features. Nutritional status was significantly associated with frailty, dehydration, ultrasound-defined sarcopenia, and calf circumference (*p* < 0.05). At univariate analysis, poorer nutritional, cognitive, and functional status, frailty, and preoperative complications were associated with higher 3-month mortality, whereas surgery within 72 h was protective.

**Discussion:**

Malnutrition, dehydration, and sarcopenia frequently coexist and are closely correlated with geriatric frailty and mortality in patients aged ≥ 65 years with PFF.

**Conclusions:**

These findings support the relevance of a comprehensive orthogeriatric assessment to better characterize biological vulnerability and guide perioperative care.

**Supplementary Information:**

The online version contains supplementary material available at 10.1007/s40520-026-03364-w.

## Introduction

Proximal femur fractures (PFFs) have become a significant global health burden due to the aging population, with over 10 million cases per year [1, 2]. Fragility fractures of the lower limbs profoundly impact patients’ lives, leading to morbidity, loss of independence in activities of daily living (ADLs), hospitalization, increased socio-economic costs, and mortality [3, 4]. These fractures are associated with early complications, such as deep vein thrombosis (DVT), pulmonary embolism (PE), pneumonia, and prolonged bed rest, as well as late complications related to the difficulty patients aged ≥ 65 years face during rehabilitation, including rapid functional decline even after brief periods of immobility [5–9]. Therefore, early surgical timing, aiming to allow for prompt rehabilitation, is a crucial factor in reducing mortality [7, 10].

Several factors contribute to frailty in patients aged ≥ 65 years, particularly nutritional and muscular status [11]. Nutritional assessment should consider both malnutrition and overnutrition, while dehydration has recently emerged as an important predictor of frailty. Regarding muscle evaluation, the ultrasound sarcopenia index (USI) reliably indicates muscle mass and function, allowing stratification of patients based on muscle architecture in both upper and lower limbs [12]. Sarcopenia is a key prognostic factor, as reduced muscle strength limits rehabilitation and decreases the likelihood of regaining pre-fracture independence.

Nutrition is a crucial determinant of health, recovery, and immune function [13]. Based on earlier studies, energetic malnutrition with protein deficit is particularly relevant in the hospital setting, reaching almost 90% in older hospitalized patients [14–16]. Nutritional screening, based on scales such as MNA and MUST, at the time of admission, is preparatory for better management of the patient from an internal medicine perspective and for the smoothest possible surgical management [17]. Dehydration is a typical finding in older patients, as patients aged ≥ 65 years have reduced perception of thirst, leading to decreased water intake [18]. This results in adverse clinical outcomes, including lethargy, confusion, acute kidney injury, pale and cold extremities, headache, impaired wound healing, and an increased risk of falls due to dizziness [19, 20]. The Clinical Dehydration Scale (CDS) is a tool that aids clinicians in assessing the severity of clinical signs of dehydration and in determining the need for oral versus intravenous fluid supplementation; however, the clinical assessment of hydration status in patients aged ≥ 65 years remains challenging and imprecise, as clinical signs, symptoms, and commonly used tests for water-loss perform poorly in this population [21].

Frailty is a multifactorial condition characterized by reduced physiological reserves, increased vulnerability to stressors, and involvement of biopsychosocial factors. The main instruments of evaluation of frailty are the Nottingham Hip Fracture Score (NHFS), Multi Prognostic Index (MPI), Charlson Comorbidity Index (CCI), and Clinical Frailty Scale (CFS) [22–25].

Malnutrition, dehydration, frailty, and sarcopenia constitute a pathophysiological cluster in which all factors are strongly interrelated and have a multifactorial effect on mortality. Although many studies have examined the impact of these individual factors on mortality after hip fracture, few have investigated their synergistic effect [10, 26, 27]. Early recognition, evaluation, and management of these factors are therefore essential for improving clinical outcomes [11].

Given the limited evidence on the interrelationships among malnutrition, dehydration, frailty, and sarcopenia, this study aimed to prospectively assess their prevalence and mutual associations in older patients with proximal femur fracture, with short-term outcomes and mortality explored as secondary endpoints.

## Materials and methods

**Fig. 1:**
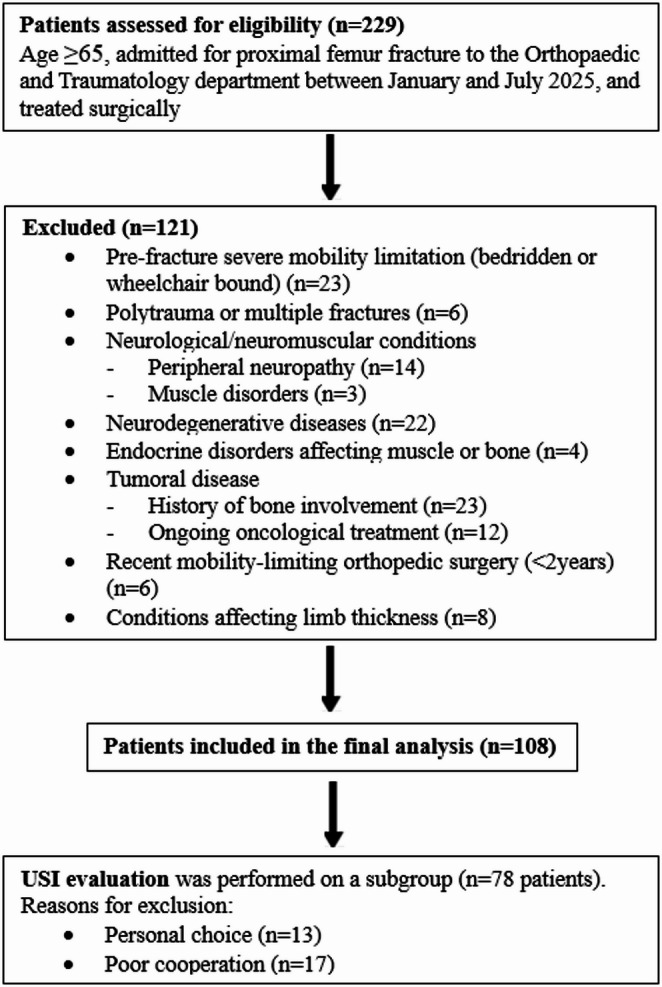
STROBE flow diagram illustrating patient selection, inclusion and exclusion criteria, and the final study population. USI: Ultrasound Sarcopenia Index

Ethical approval for the prospective observational study was granted by the local Ethics committee (CEUR –Comitato Etico Unico Regionale) with protocol code 157_2022. All consecutive patients aged ≥ 65 years admitted to the Orthopaedic and Traumatology Department for proximal femur fracture between January and July 2025 who met the inclusion criteria were prospectively enrolled. Exclusion criteria are presented in Fig. 1.

Data were collected within 48 h of admission to the hospital from the patients’ medical history, through interviews, using standardized assessment scales, and with ultrasound examination. The assessment scales that were evaluated were: MNA, MUST, SPMSQ, ADL, CCI, MPI, NHFS, CDS, Clinical Frailty Scale, and ASA score.

Nutritional status was assessed using the Mini Nutritional Assessment (MNA) and the Malnutrition Universal Screening Tool (MUST). MNA is a multidimensional tool that evaluates anthropometric measurements, dietary intake, mobility, and self-perceived health to identify malnutrition or risk of malnutrition. Scores range from 0 to 30, with established cut-offs defining normal nutritional status (24–30), risk of malnutrition (17–23.5), and malnutrition (< 17). MUST was used as a rapid screening tool. It is based on body mass index, unintentional weight loss, and the presence of acute disease. Total scores classify patients as low (0), moderate (1), or high risk (≥ 2) of malnutrition [28, 29].

Cognitive status was evaluated using the Short Portable Mental Status Questionnaire (SPMSQ), a brief screening tool assessing orientation, memory, and attention. Cognitive impairment was categorized according to the number of errors, with thresholds distinguishing normal cognition, mild, moderate, and severe impairment [30].

Functional status was assessed using the Katz Index of Activities of Daily Living (ADL), which evaluates independence in six basic self-care activities: bathing, dressing, toileting, transferring, continence, and feeding. Scores range from 0 to 6, with lower scores indicating greater functional dependency. ADL was used to reflect pre-fracture functional capacity and baseline mobility [31].

Comorbidity burden was quantified using the Charlson Comorbidity Index (CCI). This index assigns weighted scores to 19 predefined chronic conditions based on their association with mortality, resulting in a composite score that reflects the overall disease burden. Higher CCI scores indicate increased comorbidity and poorer prognosis [32].

Frailty status was determined using the Clinical Frailty Scale (CFS), a 9-point clinical judgment–based scale reflecting baseline (pre-injury) physical function, comorbidity, and cognitive status. Patients were categorized from very fit (CFS 1) to terminally ill (CFS 9), with scores ≥ 5 indicating frailty. The CFS has demonstrated prognostic value in orthopedic trauma, including the prediction of postoperative complications and mortality [24].

Short-term mortality risk in patients with hip fracture was estimated using the Nottingham Hip Fracture Score (NHFS). This validated orthopedic-specific prognostic tool incorporates age, sex, comorbidity burden, cognitive impairment, hemoglobin level on admission, living circumstances, and the presence of malignancy to estimate 30-day mortality risk. Higher NHFS scores correspond to increased risk of early postoperative mortality [25].

Hydration status was assessed using the Clinical Dehydration Scale (CDS). Although the use of CDS in older adults has been debated, particularly due to the limited specificity of clinical signs in this population, it was selected for its feasibility in the acute care setting and its previous use in hospitalized geriatric patients. A cut-off of 4 on the CDS was chosen to define dehydration, as, based on previous validation studies, scores ≥ 4 can identify patients with moderate to significant dehydration, acknowledging that CDS reflects clinical risk rather than biochemical dehydration [21].

MPI is a validated tool that was evaluated for each of the patients by the geriatric team within 24 h of admission through assessment of multiple dimensions, such as functional, cognitive, nutritional, and health statuses [22].

ASA score (American Society of Anesthesiologists) evaluates pre-anesthesia medical comorbidities by classifying patients into 6 statuses [33]. It was collected from the preoperative anesthesiologist’s report.

Limb circumferences were measured with a tape at the points of maximum circumference between shoulder and elbow (arm), elbow and wrist (forearm), hip and knee (thigh), and knee and ankle (calf).

Laboratory parameters were collected and considered in all the patients through a standardized set of exams withdrawn within 48 h that included creatinine, complete blood count, hemoglobin, sodium, potassium, and calcium levels, albumin, vitamin D, and ferritin.

The ultrasound assessment (USI) was performed using a Samsung HS60A device with a 3–14 MHz linear probe. Measurements were performed on the rectus femoris, vastus lateralis (VL), tibialis anterior, and brachioradialis muscles. Parameters included muscle thickness (Tm), fascicle length (Lf), pennation angle (θ), cross-sectional area, and the Ultrasound Sarcopenia Index (USI = Lf/Tm). VL images were acquired at the distal third of the muscle, as previously described [12, 34], with the transducer positioned perpendicularly and minimal pressure applied to avoid muscle compression. When fascicles extended beyond the field of view, ImageJ software was used to extrapolate Lf. All measurements were performed twice by a trained investigator with demonstrated inter-day reliability. Offline analysis with ImageJ included calculation of Tm, Lf, θ, and cross-sectional area. USI z-scores were derived by comparison with a young healthy control population by a previous study from Narici et al. (mean ± SD of VL: 3.70 ± 0.52) [12] and used to classify sarcopenia severity: non-sarcopenic (0–1), pre-sarcopenic (1–2), mild (2–3), full (3–4), and severe (> 4) [12]. For prevalence analysis in our cohort, USI z-scores > 2 indicated sarcopenia in both sexes.

Statistical analyses were performed using Jamovi Version 2.7.13. Continuous variables were expressed as mean ± standard deviation (SD), while categorical variables were reported as absolute frequencies and percentages. The normality of continuous variables was assessed using the Shapiro-Wilk test. For normally distributed variables, comparisons between independent groups were conducted using the independent samples t-test; otherwise, the Mann–Whitney U test was applied. Differences between categorical variables were evaluated using the Chi-square (χ²) test or, in cases of small sample sizes, Fisher’s exact test. Correlations between clinical and ultrasonographic parameters were analyzed using the Spearman correlation coefficient (ρ). Malnutrition was analyzed as a continuous variable in regression analyses. Three-month mortality was explored as a secondary, descriptive outcome of overall vulnerability. A p-value < 0.05 was considered statistically significant.

Data were anonymized, processed in compliance with GDPR, stored digitally in a secure environment, and access was restricted to authorized personnel.

## Results

The study included 108 patients, mean age 85.9 ± 6.6 years: 74 women (68.5%) and 34 men (31.5%). Table 1 presents the baseline characteristics of the study population.

**Table 1:** Baseline patients’ characteristics

Domain	Variable	Value
Study population	Patients, n Age, years Female sex, n (%)	108 85.9 ± 6.6 74 (68.5%)
Surgical data	- Surgery ≤ 24 h - Surgery ≤ 48 h - Surgery ≤ 72 h - Surgery > 72 h	25 (23%) 70 (65%) 90 (83%) 18 (17%)
	ASA score	2.3 ± 0.49
	Surgical duration, min	61 ± 25.7
Nutritional, hydration, and frailty status	At risk of malnutrition (MNA 17–23.5)	42 (39%)
	Malnourished (MNA < 17)	3 (2.7%)
	Clinical dehydration preoperatively (CDS > 4)	41 (38%)
	Frailty (CFS ≥ 5)	60 (56%)
Ultrasound-defined sarcopenia (*n* = 78)	Sarcopenia Mild sarcopenia Presarcopenia	30 (39%) 5 (6%) 2 (2%)
Hydration management	Preoperative hydration - IV - oral	32 (30%) 96 (89%)
	Postoperative hydration - IV - oral hydration	68 (63%) 94 (87%)
	Hydration status at discharge - normohydrated - dehydrated	77 (71%) 31 (29%)

ASA: American Society of Anesthesiologists Score, MNA: Mini Nutritional Assessment; CFS: Clinical Frailty Score; IV: intravenous. Data are presented as means + SD and counts and percentages as appropriate.

Other baseline characteristics, including nutritional status, prior fractures, anthropometric measurements, muscle ultrasound parameters, and pre- and post-operative complications, are presented in Supplementary Table 1.

In age- and sex-adjusted multivariable models, only a limited subset of clinical variables remained independently associated with nutritional status, hydration, sarcopenia, and frailty (Table 2).

**Table 2:** Multivariable linear regression models adjusted for age and sex showing associations between outcomes of interest

Outcome	Predictor	Beta	C.I. (95%)	*P*-value
MNA	NHFS	1.293	0.287–2.299	0.014
MNA	MUST	−2.923	−4.130 – −1.716	< 0.001
MNA	CDS	−0.894	−1.750 – −0.038	0.041
MNA	USI Z-score	−0.523	−0.907 – −0.138	0.010
CDS	NHFS	0.778	0.395–1.161	< 0.001
CDS	MNA	−0.161	−0.318 – −0.004	0.045
CFS	ADL	−0.510	−0.934 – −0.086	0.020

MNA: Mini Nutritional Assessment; NHFS: Nottingham Hip Fracture Score; CDS: Clinical Dehydration Scale; CFS: Clinical Frailty Score; ADL: Activities of Daily Living score*.*

Spearman correlation analyses revealed significant interrelationships among nutritional status, hydration, frailty, and muscle parameters (Table 3). Poor nutritional status, assessed by MNA, was significantly associated with higher frailty (CFS), increased risk of dehydration (CDS), and ultrasound-defined sarcopenia, as reflected by lower USI values and reduced calf circumference. Frailty was also significantly correlated with dehydration risk and calf circumference. Ultrasound-derived muscle parameters were strongly interrelated, particularly USI and calf circumference. No significant correlations were observed between USI Z-score and comorbidity burden, frailty, or mortality.

**Table 3:** Interrelationships between nutritional status, hydration, frailty, and sarcopenia

Variables	Malnutrition	Dehydration	Frailty	USI	Calf circumference
Malnutrition	—	0.398*	0.508*	–0.312*	0.351*
Dehydration	0.398*	—	0.331*	ns	ns
Frailty	0.508*	0.331*	—	ns	–0.331*
USI	–0.312*	ns	ns	—	–0.578*
Calf circumference	0.351*	ns	–0.331*	–0.578*	—

Spearman correlation coefficients (ρ) are reported. Malnutrition was assessed using the MNA: Mini Nutritional Assessment, dehydration was evaluated using the CDS: Clinical Dehydration Scale, while frailty was defined using the CFS: Clinical Frailty Scale. The USI: Ultrasound Sarcopenia Index was based on the vastus lateralis ns = not significant. * p < 0.05; ** p < 0.01; *** p < 0.001.

Ultrasound assessment was performed in 78 of 108 patients. The main reasons for missing USI were patient-related: lack of compliance, refusal to undergo the assessment, or being in the preoperative waiting period. No patients were excluded due to clinical instability or delirium. Baseline characteristics of patients with and without USI evaluation are summarized in *Supplementary Table 2*; no significant differences were observed in age, sex, functional status, or frailty; therefore, no formal sensitivity analysis was performed. The USI-group showed a worse nutritional status (*p* = 0.004), comorbidity burden (*p* = 0.013), and clinical signs of dehydration (*p* = 0.040).

Mortality rate in the post-operative phase was noted: 6.4% of patients died within 3 months (*n* = 7), while a 7.4% 6-month mortality rate (*n* = 8) was recorded.

A univariate analysis was performed with results shown in Table 4 based on factors that were assumed to influence the mortality rate at 3 months. The need for intravenous hydration (*p* = 0.030) and reduced or absent oral intake (*p* < 0.001) during hospitalization were more frequently observed among patients who died within three months.

**Table 4:** Exploratory univariate analysis of the factors that could affect the mortality rate at 3 months

	O.*R*.	C.I. (95%)	*P*-value
Sex	1.333	0.300–5.942	0.704
Age	1.14	0.981–1.321	0.088
MNA	0.513	0.346–0.760	**< 0.001**
SPMSQ	1.513	1.088–2.104	**0.014**
ADL	0.659	0.451–0.961	**0.030**
CCI	1.037	0.619–1.739	0.888
NHFS	1.786	0.776–4.114	0.172
MPI	0.159	0.013–1.910	0.147
MUST	1.207	0.477–3.058	0.780
CFS	1.811	1.010–3.238	**0.045**
CDS	0.680	0.395–1.17	0.162
Preop. Compl.	8.478	1.541–46.618	**0.014**
Delirium preop	6.333	1.010–39.705	0.049
Surgery <72 h	0.163	0.036 - 0.727	**0.017**
USI vastus lateralis	2.394	0.754–7.600	0.138

Given the limited number of deaths, results should be interpreted as exploratory and hypothesis-generating. MNA: Mini Nutritional Assessment; SPMSQ: Short Portable Mental Status Questionnaire; ADL: Activities in Daily Living; CCI: Charlson Comorbidity Index; NHFS: Nottingham Hip Fracture Score; MPI: Multidimensional Prognostic Index; MUST: Malnutrition Universal Screening Tool; CFS: Clinical Frailty Scale; CDS: Clinical Dehydration Scale; Preop. Compl: preoperative complications

Given the limited number of deaths at three months (*n* = 7), multivariable models including multiple predictors were not performed to avoid overfitting.

Overall, these findings indicate that malnutrition, dehydration, frailty, and sarcopenia are closely interrelated in older patients with proximal femur fracture, but only specific components retain independent associations when accounting for age and sex.

## Discussion

Frailty, malnutrition, and dehydration are interrelated conditions in patients aged ≥ 65 years with proximal femur fractures, and their management is essential for improving prognosis in vulnerable patients, as already highlighted by several studies [3, 10].

The study population consisted of 108 consecutive older patients with proximal femoral fractures, with a mean age of 85.9, comparable to that reported in the literature [4]. Multidimensional assessment at hospital admission revealed a condition of generalized geriatric vulnerability, characterized by the coexistence of nutritional, functional, and muscular impairments of varying severity [27].

Analysis of clinical and functional questionnaires documented mean scores consistent with moderate frailty and risk of malnutrition. In particular, the MNA showed mean results that indicated that a significant amount of patients were at risk of malnutrition and the CFS recorded mean values that showed a condition of widespread clinical frailty, while the CDS showed a mean score suggesting the presence of preoperative clinical signs of dehydration in a substantial portion of patients, consistent with previous reports by Ekman et al. describing a preoperative clinically assessed dehydration prevalence of 20.4% [19]. Pre-fracture autonomy assessed by the ADL scale showed partial loss of independence. Prognostic and comorbidity indices, including CCI, MPI, and NHFS, confirmed an elevated level of clinical complexity and an increased risk of adverse outcomes. Nutritional status assessed by MNA showed stronger associations with frailty, dehydration, and sarcopenia than MUST. This difference likely reflects the multidimensional nature of MNA, which includes dietary, functional, and psychosocial components, making it more suitable for identifying biological vulnerability in older orthopedic patients and capturing chronic nutritional deficits.

Clinical assessment of hydration in patients aged ≥ 65 years remains challenging, as common measures (e.g., dry mouth, urine color) are unreliable [20]. In this study, CDS served as a proxy for dehydration risk rather than a definitive diagnostic tool. Dehydration correlated with poorer MNA scores, reduced ADL, and higher comorbidity burden, suggesting a predominant clinical-functional mechanism rather than structural renal impairment.

Multivariable models adjusted for age and sex showed that malnutrition was associated with frailty, dehydration, and lower muscle mass; hydration status was influenced by frailty, MNA, and sex; and frailty was mainly determined by pre-fracture functional status. Cognition (SPMSQ), comorbidities (CCI), MPI, and age had no independent effects. These results highlight that only a subset of interrelated parameters exerts independent influence on patient vulnerability.

Malnutrition and clinically assessed dehydration were associated with higher short-term mortality, consistent with previous reports [11, 13, 18–20], though the limited number of events precluded multivariate analyses. Surgery within 72 h was associated with lower 3-month mortality (*p* = 0.017); however, 17% of patients underwent surgery after 72 h, reflecting the need to balance early surgery with optimization of complex, multimorbid patients [6, 27, 35]. Statistical analyses revealed multiple interconnections between nutritional status, frailty, dehydration, and sarcopenia in our cohort.

USI evaluation revealed reduced fascicle length and increased pennation angle. These measurements strongly correlated with anthropometric muscle measures, confirming USI’s sensitivity in detecting sarcopenic remodeling [28, 34]. USI Z-score was not independently associated with frailty, comorbidity, or short-term mortality, indicating that ultrasonographic sarcopenia may reflect muscle quality independently of age or disease burden. Sarcopenia’s prevalence was high, affecting over one-third of patients assessed. Ultrasound-derived muscle measurements also highlighted differences between upper and lower limb muscle trophism. While brachioradialis thickness and limb ratios reflected upper limb muscle status, the tibialis anterior index provided insight into lower limb muscle mass. Upper arm circumference also showed a significant association with malnutrition (*p* = 0.017).

Regarding sex-related differences, males had a higher frequency of preoperative complications (*p* = 0.003; Exp(B) = 0.208; OR ≈ 1.23), suggesting a higher trend toward vulnerability and higher comorbidity burden in males [23, 36, 37].

This study has several limitations that need to be addressed. First, the small number of deaths at three months restricted multivariate regression analyses, so associations should be interpreted as observational trends. Second, while multivariable models were used to examine the associations between nutritional status, dehydration, frailty, and muscle parameters, residual confounding cannot be excluded. Third, ultrasound assessment was performed in 78 of 108 patients, which may limit the generalizability of sarcopenia-related findings. However, missing ultrasound data were mainly due to patient-related factors (e.g., lack of compliance or preoperative timing) rather than clinical instability, and baseline comparisons between patients with and without ultrasound assessment did not reveal significant differences in age, sex, functional status, or frailty. Finally, USI Z-scores are compared to a young, healthy population rather than age-matched controls. While this provides insight into muscle quality and sarcopenic remodeling, it may not fully account for the natural aging process in older adults. Despite these limitations, this study provides a novel, integrated perspective by simultaneously evaluating nutritional status, clinical dehydration, frailty, and ultrasound-derived muscle architecture in the acute setting of proximal femur fracture, moving beyond single-factor risk assessment and highlighting their synergistic roles.

## Conclusion

In conclusion, this prospective study highlights the combined role of malnutrition, dehydration, frailty, and ultrasound-defined muscle impairment in shaping biological vulnerability in patients aged ≥ 65 years with proximal femur fractures. Early identification of this vulnerability cluster may support more targeted nutritional, hydration, and rehabilitation strategies beyond surgical timing alone.

## Supplementary Information

Below is the link to the electronic supplementary material.

Supplementary Material 1

## Data Availability

Additional data supporting the findings of this study are available from the corresponding author upon reasonable request.
