# Supplementary material for: Interrelationships between malnutrition, dehydration, frailty, and sarcopenia in older adults with proximal femur fractures: a prospective observational study
Source: Aging Clin Exp Res. 2026 Apr 13;38(1):136. doi: 10.1007/s40520-026-03364-w (PMC13194301; doi:10.1007/s40520-026-03364-w)
Supplement: Supplementary file 1 — Supplementary Material 1 [file 40520_2026_3364_MOESM1_ESM.docx]

Supplementary Table 1.

| Domain | Variable | Value |
| --- | --- | --- |
| Osteoporosis therapy at admission | Antiresorptive/anabolic monoclonal antibodies  Vitamin D and calcium  Bisphosphonates | 9 (8%)  38 (35%)  3 (2.7%) |
| Biochemical parameters at admission | - Albumin, g/dL - Vitamin D, ng/mL - Hemoglobin, g/dL - Ferritin, ng/mL | 3.55 ± 0.37  21.7 ± 11.2  11.9 ± 1.8  180.9 ± 370 |
| Prior fragility fractures | Prior fragility fracture   - Vertebral - Femoral - Humeral - Wrist | 28 (26%)  11 (10%)  8 (7%)  11(10%)  2 (2%) |
| Anthropometric measures | Forearm circumference, cm  Upper arm circumference, cm  Thigh circumference, cm  Calf circumference, cm | 19.9 ± 2.3  24.5 ± 3.6  38.8 ± 4.9  28.9 ± 3.8 |
| Muscle ultrasound assessment (n = 78) | Rectus femoris USI  Rectus femoris Z-score  Vastus lateralis USI  Vastus lateralis Z-score  Rectus femoris PA/MT  Vastus lateralis PA/MT  Rectus femoris Lf/PA  Vastus lateralis Lf/PA | 3.18 ± 0.88  −0.98 ± 1.70  3.37 ± 0.76  −0.64 ± 1.46  6.49 ± 2.68  6.40 ± 2.50  0.50 ± 0.10  0.45 ± 0.09 |
| Upper and lower limb muscle indices | Brachioradialis thickness, cm  Upper-to-lower limb ratio  Normalized upper-to-lower limb ratio  Tibialis anterior trophism index | 1.20 ± 0.24  2.62 ± 0.61  1.34 ± 0.28  0.131 ± 0.03 |
| Hydration management and complications | Preoperative complications   - Acute renal failure - Delirium - Pneumonia | 28 (26%)  61 (56%)  30 (28%)  11 (10%) |
|  | Post-op complications   - Acute renal failure - Delirium - Electrolyte disorders | 24 (22%)  44 (41%)  36 (33%)  72 (67%) |

*Supplementary Table 1. Baseline characteristics of the study population. Data are presented as mean ± standard deviation for continuous variables or as counts and percentages for categorical variables. Data are presented on the number of patients with a history of prior fragility fractures, as well as the number of fractures for each type of fragility fracture. Muscle ultrasound assessments were performed in a subset of 78 patients. MNA: Mini Nutritional Assessment; CDS: Clinical Dehydration Score; CFS: Clinical Frailty Scale; USI: Ultrasound Sarcopenia Index; PA: Pennation Angle; MT: Muscle Thickness; Lf/PA: Fiber Length / Pennation Angle ratio. The upper-to-lower limb ratio represents the ratio between upper limb (brachioradialis) and lower limb (vastus lateralis) muscle thickness, providing an estimate of the relative distribution of muscle mass. The normalized upper-to-lower limb ratio is adjusted for body size to allow inter-individual comparison. The tibialis anterior trophism index reflects distal lower limb muscle trophism and is given by the ratio between the thickness and the cross-sectional area of the tibialis anterior.*

*Supplementary Table 2*

| Variable | USI (n=78) | Not USI (n=30) | p-value |
| --- | --- | --- | --- |
| Age, years | 84 (82-88) | 86 (82-90) | 0.531 |
| Female sex, n (%) | 54 (69%) | 20 (66%) | 0.797 |
| ADL (pre-fracture) | 6 (4-6) | 6 (3-6) | 0.853 |
| SPMSQ | 2(2-3) | 2(2-4) | 0.053 |
| MNA | 23(21.25-25.5) | 25 (23-28) | 0.004 |
| CCI | 5 (4-6) | 5(4-5) | 0.013 |
| CDS | 2 (1-2) | 1 (0-2) | 0.040 |
| MPI | 1 (1-1) | 1 (1-1) | 0.371 |
| CFS | 5 (3-6) | 5 (3-6) | 0.855 |

*Supplementary Table 2: Baseline characteristics of patients who underwent USI evaluation vs those who did not. Continuous variables are reported as median (IQR) and compared using the Mann–Whitney U test; categorical variables are presented as counts and percentages and are compared using the χ² test. ADL: Activities of Daily Living, SPMSQ: Short Portable Mental State Questionnaire, MNA: Mini Nutritional Assessment, CCI: Charlson Comorbidity Index, CDS: Clinical Dehydration Scale, MPI: Multidimensional Prognostic Index.; CFS: Clinical Frailty Score*
